# Disordered Eating Attitudes and Their Association with Age, BMI, Stress, and Diet in College Students

**DOI:** 10.3390/ijerph21060766

**Published:** 2024-06-13

**Authors:** Niliarys Sifre, Rianna Deringer, Lukkamol Prapkree, Cristina Palacios

**Affiliations:** Dietetics and Nutrition Department, Robert Stempel College of Public Health & Social Work, Florida International University, Miami, FL 33174, USA; nsifr001@fiu.edu (N.S.);

**Keywords:** disordered eating attitudes, diet, snacks, stress, college students, overweight/obesity

## Abstract

Objective: College students are at risk of disordered eating, particularly students with overweight/obesity and with higher stress, but little is known about how disordered eating may be related to diet. This study evaluated the associations between the Disordered Eating Attitudes Scale (DEAS) and age, BMI, stress, and diet. Methods: This is a secondary analysis of the baseline data in participants from the Snackability Trial. Participants completed a questionnaire on socio-demographics, DEAS, and snacking, self-reported their weight and height (to calculate BMI), and completed two 24 h non-consecutive dietary recalls (to calculate diet quality using HEI-2015 and snack quality score using an algorithm developed by our group). Associations between variables were assessed with Spearman correlations. Results: A total of 140 participants completed all assessments. The median age was 21.0 and the median BMI was 28.5 kg/m^2^ (43.7% had overweight and 41.5% had obesity). A total of 86.4% were females, 41.4% were white, 51.4% were low-income, and 30.7% were Hispanic/Latino. The total DEAS and the subscale ‘Relationship with food’ were positively correlated with stress and BMI (*p* < 0.05) but inversely correlated with HEI-2015 (*p* < 0.05). The subscales ‘Restrictive and compensatory behaviors’ and ‘Concern about food and weight gain’ were also positively correlated with stress (*p* < 0.001). Conclusion: College students with higher disordered eating attitudes also had higher stress and BMI but poorer diet quality. Interventions may be needed for this group to manage stress and improve weight and diet quality, as well as promote awareness about disordered eating attitudes.

## 1. Introduction

Disordered eating (DE) is an umbrella term for irregular eating behaviors including a preoccupation with a certain food group, binging, or purging, among other behaviors [1]. It has been estimated that 5–20% of individuals have symptoms of disordered eating [2,3], and this could impact their overall health. Therefore, it is important to understand the factors that could lead to disordered eating. This can be achieved by evaluating factors associated with the Disordered Eating Attitudes Scale (DEAS), a scale that includes abnormal beliefs, thoughts, feelings, behaviors, and relationships to foods, which often precede a diagnosis of disordered eating [4].

There is evidence that age, gender, race, ethnicity, and economic status are related to disordered eating attitudes in college students [5]. Also, individuals who are younger, women, white, Hispanic, or from low socio-economic backgrounds tend to have higher disordered eating attitudes [6,7,8,9,10]. Stress and anxiety, which are particularly important during the college years in students [11], have been also associated with disordered eating attitudes [12]. Sources of stress and anxiety in college include selecting a major, exams, assignment deadlines, demands of challenging professors, planning for the future, and for some, the transition from financial dependence to independence [13]. A few studies among college students have found a higher level of disordered eating attitudes during the examination stress period [14] or at the end of the freshman year, which was also found to be related to a high perceived stress level [5]. A higher BMI has also been related with greater disordered eating attitudes among black US college students [15], among female college students from Saudi Arabia [16], and among female college students from Kuwait [17]. This could be related to higher levels of body dissatisfaction and concerns with weight [9,18]. Disordered eating attitudes could also be associated with certain dietary patterns; a study among 1645 US college students with food addiction and eating disorder symptoms found a lower intake of vegetables and higher consumption of saturated fat and added sugar [19]. Snacking, particularly abnormal snacking patterns, could be characterized as a symptom of disordered eating [4]. Although snacking is very common in the US [20], particularly among college students [21,22,23], there are very limited studies showing how snacking is related to disordered eating attitudes, and to our knowledge, no studies have associated this with diet quality.

Therefore, the purpose of the present secondary analysis was to expand our current knowledge on the association between disordered eating attitudes and age, stress, BMI, snacking patterns, snack quality, and diet quality among college students in the US. The data analyzed in this study came from the Snackability Trial, which was a two-arm, 12-week randomized controlled trial that tested the efficacy and usability of Snackability app [24] for improving the quality of snacks.

## 2. Methods

This is a secondary analysis of the baseline visit of the Snackability trial [25]. This was a two-arm, 12-week randomized controlled trial that tested the efficacy and usability of a newly developed smartphone application (*Snackability*) [24] for improving the quality of snacks. The baseline visit was completed between June 2020 and March 2021.

### 2.1. Sample

College students from different US colleges were eligible for participation if they were 18–24 years old, owned a smartphone with Android or iOS platforms, had access to an internet connection to use the app, and were willing to participate in a clinical trial for 3 months. Participants were excluded if they were currently enrolled in a weight loss and/or nutrition program, were nutrition students, were taking any medications known to influence weight, and were pregnant or breastfeeding.

### 2.2. Recruitment and Screening Process

All study procedures were approved by the Institutional Review Board of FIU. Written informed consent was obtained from all participants prior to study commencement. Participants were recruited mainly through emails, social media, and referrals from friends. Interested students were first asked to complete an online screening checklist to confirm that they qualified to participate in the study via Qualtrics, a secured web-based survey. If they qualified, they were automatically allowed to continue with the online consent form. Once they signed, they were automatically allowed to continue with the baseline online questionnaire and other assessments.

### 2.3. Assessments

(1). General questionnaire: participants completed an online questionnaire (via Qualtrics) with the following information:

Socio-demographics: Participants were asked about their age (in years), gender (male, female, or other), race/ethnicity (white, black, Asian, other, Hispanic/Latino), household income (<USD 50,000 or USD 50,000–USD 75,000 or USD 75,000–USD 100,000 or >100,000), and field of study.

Stress: The level of stress over the past week was assessed using a validated scale (stressometer) [26], in which students were asked to select a number from 0 (no stress) to 10 (extreme stress).

Disordered Eating Attitudes Scale (DEAS): Participants were asked to complete a 25-item validated questionnaire to assess disordered eating attitudes [4,27]. This questionnaire included several questions that used a Likert-based scale of questions as well as yes/no answers to several of the premises. The total score ranges from 37 to 190, in which a higher DEAS indicates a greater DEA. It also provides the score for each of the 5 subscales: Relationship with food, Concern about food and weight gain, Restrictive and compensatory practices, Feelings toward eating, and Idea of normal eating.

Snack intake: Participants completed a question about the frequency of snacks consumed per day and per week.

(2). Dietary recalls: Participants were also asked to complete three non-consecutive 24 h dietary recalls using the Automated Self-Administered 24 h (ASA24) Dietary Assessment Tool, version 2020 [28] The mean of the 24 h recalls was used in all analyses. The diet quality was assessed using the Healthy Eating Index (HEI)-2020 total score and component scores for each participant [29] using the simple HEI scoring algorithm method in ASA24 [30]. The HEI-2020 total score consists of the sum of the following components: 9 adequacy components (total vegetables, greens, beans, total fruits, whole fruits, whole grains, dairy, total protein foods, seafood, plant proteins, and fatty acids) and 4 moderation components (refined grains, sodium, saturated fats, and added sugars). Total scores range from 0 to 100, in which a higher score is a diet that is more aligned with the Dietary Guidelines for Americans (DGA) 2020–2025 and, therefore, healthier [29]. The snack quality was assessed from snacks identified from the 24 h recalls completed in ASA24. First, for each snack consumed, the type, number, and serving size were recorded. Then, using the scoring algorithm developed for the Snackability app [24], the snack quality score was calculated. Briefly, this score was based on the USDA Smart Snack Guideline [31]. It considers the first ingredient for each snack (i.e., fruit, vegetable, nuts, seeds, etc.), the nutrient standard by portion size (amount of sugar, sodium, saturated fat, etc.), and the level of processing of that snack (number of additives, preservatives, etc.). The total snack quality scores range from −1 to 11, in which a higher score is a snack that is more compliant with the guidelines and, therefore, healthier.

(3). Body weight and height: Participants were also asked to weigh themselves at home using a standardized protocol with written and video instructions. Participants were instructed to weigh themselves in the morning, after voiding and before eating or drinking, wearing only light underclothing and barefoot, and to place the scale on a hard and flat surface floor. Before this, participants were asked to calibrate the scale following the instructions shown in the video. A scale was shipped to those who reported not having a scale. The participants reported their weight with 1 decimal in kg or pounds (lb) in duplicate and their height in inches through Qualtrics. The body mass index was calculated as kg/m^2^.

### 2.4. Statistical Plan

Descriptive statistics included the frequency and percent for categorical variables and median and percentiles (25th and 75th) for continuous variables. The associations between the total DEAS and the DEAS subscales with age, income, stress level, BMI, snack intake, snack score, and HEI-2015 scores were assessed with Spearman correlations, since these variables were not normally distributed. DEAS scores were also compared by gender and race/ethnicity using a Mann–Whitney U-Test. Analyses were performed using SPSS software version 28.0, and statistical significance was set at *p* < 0.05.

## 3. Results

The baseline questionnaire was completed by 140 students. The median age was 21.0 years, most were females (86.4%), white (41.4%), with a household income of <USD 50,000 (51.4%), and 30.7% were Hispanic/Latino (Table 1). The median BMI was 28.5 kg/m^2^, with 14.8% having a healthy weight, 43.7% being overweight, and 41.5% having obesity. The median stress level was 7.0 (which is considered a medium stress level), the median DEAS was 86.0 (which is considered a medium DEAS level), and the median snack intake was 2.0 snacks per day.

Table 2 shows the correlation between the DEAS and age, stress, and BMI. No significant correlations were observed between the DEAS and age. Total DEAS, subscale 1 (Relationship with food), and subscale 3 (Restrictive and compensatory behaviors) were positively correlated with stress (*p* < 0.001). Total DEAS and subscale 1 were also positively correlated with BMI (*p* < 0.05). The DEAS values were similar by gender or race/ethnicity (results not shown).

Table 3 shows the correlation between the DEAS and snack intake, snack score, and HEI-2015. Total DEAS or its subscales were not significantly correlated with snack intake or snack quality score. However, total DEAS (*p* = 0.033) and subscale 1 (*p* = 0.026) were inversely correlated with the HEI-2015 score (diet quality).

## 4. Discussion

The present study found a higher level of disordered eating attitudes with higher stress and BMI and lower diet quality (HEI-2015) in this diverse sample of 140 US college students, in which most were categorized as overweight or having obesity. No associations were found between disordered eating attitudes and age, gender, race/ethnicity, or snacking.

With respect to stress, not only was the total DEAS associated with higher stress levels, so were the subscales ‘Relationship with food’, ‘Concern about food and weight gain’, and ‘Restrictive and compensatory behaviors’, which is consistent with other studies. For example, disordered eating attitudes were positively associated with psychological distress in studies among 1051 college students from China and 66 young adults from eating disorder clinics in Washington, US [32,33], and with higher anxiety levels among 1040 college students from Northeastern University [14]. High perceived stress and anxiety have also been associated with increased unhealthy dietary behaviors in 393 college students from Korea [34] and in 1055 college students (311 male and 744 female) from Spain [35]. Furthermore, certain disordered eating behaviors and mealtime cognitions (e.g., a worry about gaining weight) have been associated with continued anxiety before, during, and after mealtimes in US patients with eating disorders [33]. Lastly, a lack of confidence in coping mechanisms was more likely to encourage negative eating attitudes, especially when associated stressors were introduced to their everyday lives, in 150 US college students majoring in psychology [36]. These results may be explained in part by the transition that students go through from a more structured environment, such as high school and the home, to the university/work environment. In addition, this new environment increases stress in college students [11]. Also, a study among 178 students from New Zealand found that those who suffered from homesickness had lower measures of internal locus of control [37]. Having a low internal locus of control perception, in play with other factors, is a common risk for developing disordered eating attitudes [38].

The present study also found that students with a higher total DEAS score and score on the DEAS subscale ‘Relationship with food’ had a higher BMI. This is consistent with a few other studies among college students. For example, a study among 191 black female college students from a mid-Atlantic public university found that participants with a higher BMI showed less body appreciation and greater disordered eating attitudes [15]. Similarly, a study conducted among 550 female college students in Saudi Arabia found that those with a higher BMI also had more disordered eating attitudes (52.3%) compared to those that were underweight (35.2%; *p* < 0.01) [16]. Lastly, a study among 1147 female college students from Kuwait found that those with overweight or obesity had higher disordered eating attitudes (52.1%) compared to those with normal weight or underweight (38.8%; *p* < 0.01) [17]. A higher BMI may be associated with higher levels of body dissatisfaction and concerns with weight, which is also associated with unhealthy behaviors around food to control weight or shape [9,18].

In contrast, the present study also found that a lower total DEAS and score on the subscale ‘Relationship with food’ were found among those with a higher HEI-2015 score, indicating a better diet quality, but no associations were found with snacking or snack quality score. To our knowledge, there are only a handful of studies that have evaluated the associations between disordered eating attitudes and diet or snacking. In a study among 1575 college students from a Midwestern university, those with healthier eating attitudes also had a higher diet quality [39]. Another study among 151 adolescents with type 1 diabetes found that those who were at risk of disordered eating had poorer diet quality [40]. Two studies in adolescents from the US found that those with loss-of-control eating episodes (a specific eating disorder) were more likely to consume snacks than those without this disorder [41,42]. Because snacks are often consumed out of convenience and often mindlessly at the same time as other tasks are being finished at school, work, home, etc. [43], one could argue that mindless eating could affect the recall of snack consumption during the day, and they could be disregarded as important or influential when answering the DEAS questionnaire. It has been determined that mindful eating requires self-monitoring and an active processing of what is being consumed [44] Thus, more studies are needed to understand if disordered eating attitudes are related to snacking in college students and in other age groups.

The present study had a few limitations and strengths that should be considered. The study was conducted amidst the COVID-19 pandemic, which may have influenced the college students’ living and studying environment, and therefore, it could have impacted the disordered eating attitudes. In fact, studies conducted during or after the pandemic found a significant increase in snacking [45,46] and a trend for an increase in eating disorders and disordered eating attitudes during this time [47,48]. However, due to the lack of studies evaluating these associations during such an important stage of life, this study expands our current knowledge on the association between disordered eating attitudes and diet and other factors. Also, over 86.4% of our participants were females, >50% were low-income, and >85% had overweight or obesity; the results could be different among other college students. Furthermore, it is important to publish these types of studies in public health journals, given the impact of disordered eating on the population and the health care system [49]. The limitations of the present study could be addressed in future research by expanding the sample size to achieve a more diverse sample. Future research should also evaluate variables that could have a mediating effect. The strengths of our study include the diversity of the students in terms of race and ethnicity, as students were recruited online from various universities in the US, the use of three dietary recalls to evaluate diet quality using the HEI-2015 score, and the use of a validated questionnaire to assess disordered eating attitudes.

In conclusion, this analysis showed that college students with higher disordered eating attitudes also had higher stress levels and BMIs but poorer diet quality. Based on these findings, colleges may need to develop interventions for college students, particularly those with overweight and obesity, to manage stress and improve weight and diet quality, as well as promote awareness for students with high levels of disordered eating attitudes.

## Figures and Tables

**Table 1:** Socio-demographic characteristics of the sample.

Variable	Median (25th and 75th)
Age (years)	21.0 (20.0 and 22.0)
Gender	
Female	121 (86.4)
Male	19 (13.6)
Race	
White	58 (41.4)
Black	15 (10.7)
Asian	15 (10.7)
Mixed	4 (2.9)
Ethnicity	
Hispanic/Latino	43 (30.7)
Non-Hispanic	97 (69.3)
Household income	
<USD 50,000	72 (51.4)
USD 50,000–USD 75,000	25 (17.9)
USD 75,000–USD 100,000	17 (12.1)
>USD 100,000	26 (18.6)
Stress level	7.00 (6.00 and 8.00)
BMI (kg/m^2^)	28.5 (26.2, 33.8)
DEAS ^1^	86.0 (73.0 and 102.0)
Snack intake per day	2.0 (2.0 and 3.0)
Snack frequency per week	6.0 (5.0 and 7.0)
Snack Score	2.65 (2.4 and 2.8)
HEI-2015 score	53.5 (46.13 and 61.87)

^1^ Disordered Eating Attitudes Scale.

**Table 2:** Spearman correlation between total DEAS and subscales of DEA with age, stress, and BMI.

Scale	Age		Stress		BMI	
	R	*p* Value	R	*p* Value	R	*p* Value
Relationship with food	−0.027	0.754	0.352	**<0.001**	0.288	**0.001**
Concern about food and weight gain	0.035	0.690	0.194	**0.023**	0.027	0.751
Restrictive and compensatory behaviors	−0.079	0.360	0.327	**<0.001**	0.012	0.894
Feelings toward eating	0.116	0.178	0.116	0.177	0.108	0.210
Idea of normal eating	0.075	0.387	0.022	0.799	0.077	0.274
Total DEAS	−0.024	0.777	0.317	**<0.001**	0.193	**0.025**

Bold just highlights the significant values.

**Table 3:** Spearman correlations between total DEAS and subscales of DEA with snack intake, snack score, and HEI-2015.

	Snack Intake per Day		Snack Intake per Week		Snack Score		HEI-2015	
	R	*p* Value	R	*p* Value	R	*p* Value	R	*p* Value
Relationship with food	0.148	0.086	−0.002	0.984	−0.108	0.234	−0.191	**0.026**
Concern about food and weight gain	0.032	0.713	−0.114	0.186	0.081	0.376	−0.042	0.631
Restrictive and compensatory behaviors	0.116	0.179	−0.014	0.992	−0.064	0.485	−0.017	0.846
Feelings toward eating	−0.023	0.789	−0.096	0.868	−0.111	0.223	−0.056	0.518
Idea of normal eating	0.044	0.614	−0.009	0.913	0.092	0.315	−0.147	0.087
Total DEAS	0.105	0.223	−0.040	0.643	−0.074	0.415	−0.183	**0.033**

Bold highlights the significant values.

## Data Availability

Data are available upon request.
